# A nomogram and risk classification system forecasting the cancer-specific survival of lymph- node- positive rectal cancer patient after radical proctectomy

**DOI:** 10.3389/fonc.2023.1120960

**Published:** 2023-02-01

**Authors:** Chonghan Zhong, Houqiong Ju, Dongning Liu, Penghui He, Daqiang Wang, Hongxin Yu, Weijie Lu, Taiyuan Li

**Affiliations:** Department of General Surgery, The First Affiliated Hospital of Nanchang University, Jiangxi, Nanchang, China

**Keywords:** nomogram, risk stratification, lymph node positive, cancer-specific survival, SEER, rectal cancer

## Abstract

**Background:**

The aim of the study was to develop and validate a nomogram for predicting cancer-specific survival (CSS) in lymph- node- positive rectal cancer patients after radical proctectomy.

**Methods:**

In this study, we analyzed data collected from the Surveillance, Epidemiology, and End Results (SEER) database between 2004 and 2015. In addition, in a 7:3 randomized design, all patients were split into two groups (development and validation cohorts). CSS predictors were selected *via* univariate and multivariate Cox regressions. The nomogram was constructed by analyzing univariate and multivariate predictors. The effectiveness of this nomogram was evaluated by concordance index (C-index), calibration plots, and receiver operating characteristic (ROC) curve. Based on the total score of each patient in the development cohort in the nomogram, a risk stratification system was developed. In order to analyze the survival outcomes among different risk groups, Kaplan–Meier method was used.

**Results:**

We selected 4,310 lymph- node- positive rectal cancer patients after radical proctectomy, including a development cohort (70%, 3,017) and a validation cohort (30%, 1,293). The nomogram correlation C-index for the development cohort and the validation cohort was 0.702 (95% CI, 0.687–0.717) and 0.690 (95% CI, 0.665–0.715), respectively. The calibration curves for 3- and 5-year CSS showed great concordance. The 3- and 5-year areas under the curve (AUC) of ROC curves in the development cohort were 0.758 and 0.740, respectively, and 0.735 and 0.730 in the validation cohort, respectively. Following the establishment of the nomogram, we also established a risk stratification system. According to their nomogram total points, patients were divided into three risk groups. There were significant differences between the low-, intermediate-, and high-risk groups (p< 0.05).

**Conclusions:**

As a result of our research, we developed a highly discriminatory and accurate nomogram and associated risk classification system to predict CSS in lymph-node- positive rectal cancer patients after radical proctectomy. This model can help predict the prognosis of patients with lymph- node- positive rectal cancer.

## 1 Introduction

Rectal cancer is one of the most common gastrointestinal malignancies, and adenocarcinomas are the most common pathological types, worldwide (1). Lymph node metastasis is the most common route of metastasis in rectal cancer (2–4). The rate of lymph node metastasis is as high as 15% even in patients with stage T1 (tumor confined to the submucosa) (5). Positive lymph node is an important factor affecting the prognosis of rectal cancer patients and is also an important basis for the selection of postoperative adjuvant therapy. Studies have shown that patients who are lymph node positive have a higher recurrence rate, a lower survival rate, and a poorer prognosis (6, 7). As a result, a prognostic model needs to be developed for patients with lymph- node- positive rectal cancer.

A nomogram is a statistical prediction tool that provides prognostic information about an individual’s health (8). The nomogram consists of basic variables such as individual basic information, tumor pathology, and treatment modalities (9). The nomogram takes advantage of the numerical strength of the data to facilitate a probabilistic analysis of tumor-related risk factors when compared to other prediction tools. Up to now, many nomograms regarding the prognosis of rectal cancer had been established (10, 11). However, nomograms predicting CSS in lymph- node- positive rectal cancer patients are yet to be fully developed and validated.

The data used in our study were obtained from the National Cancer Institute’s Surveillance, Epidemiology, and End Results (SEER) database. The database data include patient information, pathological information, and social information, providing important data and evidence to support medical research (12, 13). We obtained clinical and pathological characteristics of patients who had lymph- node- positive rectal cancer from the SEER database between 2004 and 2015. Identifying risk factors to establish a practical nomogram for predicting lymph- node- positive rectal cancer patients at 3- and 5- year CSS. Furthermore, the study evaluated the performance of the nomogram and its applicability according to an internal validation process.

## 2 Patients and method

### 2.1 Patient data collection

Data for these patients come from the SEER database, which provide a good representation of the epidemiology and cancer statistics of the US population. Using SEER*Stat 8.4.0.1 software, we extracted clinically relevant data of patients diagnosed with rectal cancer from 2004 to 2015 from SEER Research Plus Data.17 Registries, Nov 2021 Sub (2000–2019). Data included baseline demographics, tumor characteristics, treatment information, diagnostic staging, and survival time.

**Figure 1:**
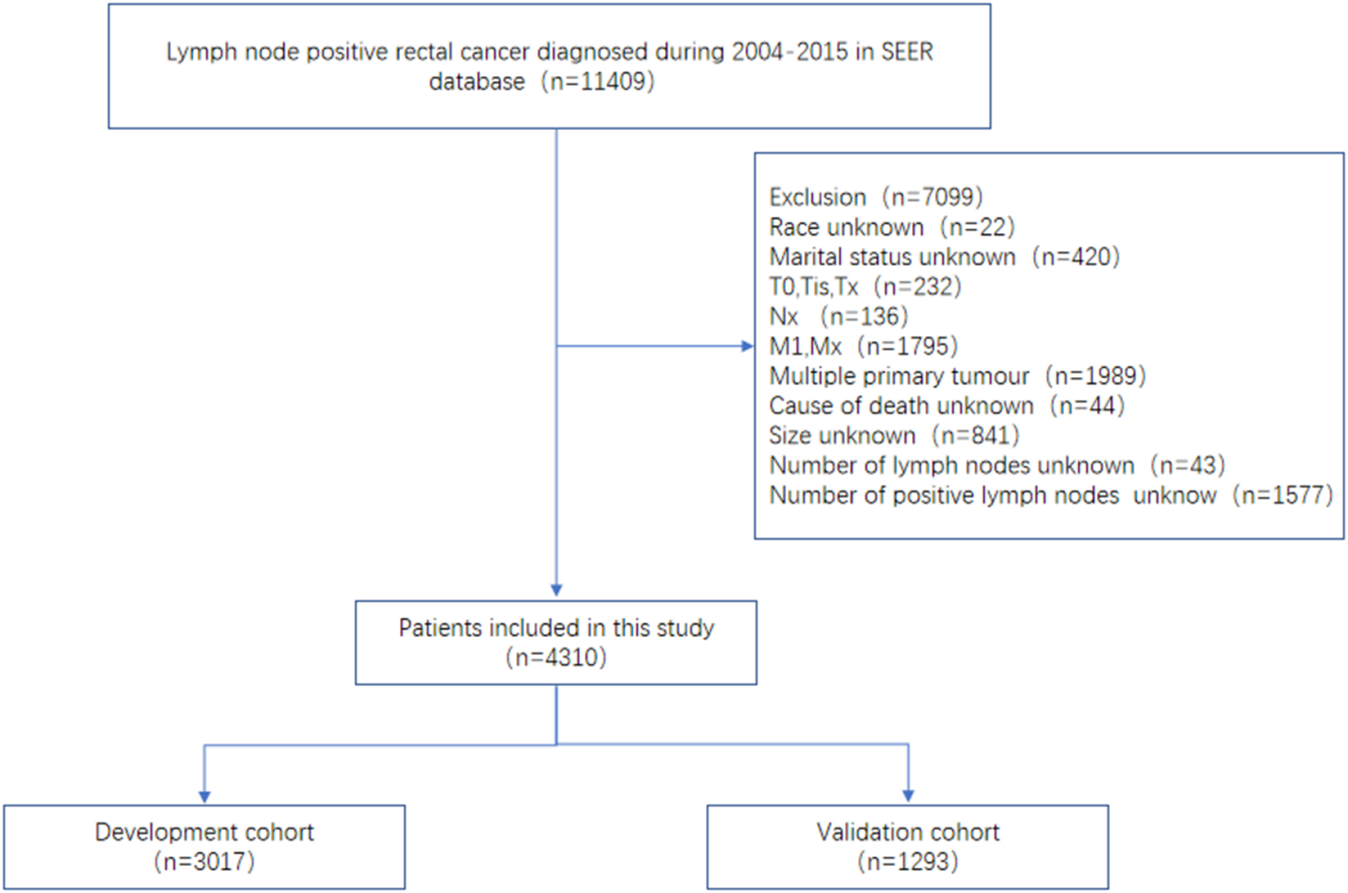
Flow diagram of the rectal cancer patients with development and validation cohorts.

Data inclusion relies on the following inclusion criteria: (a) the disease was diagnosed between 2004 and 2014; (b) the number of positive lymph nodes is not 0; (c) surgical procedures to determine positive lymph nodes include anterior resection, Hartmann’s operation, low anterior resection (LAR), and trans-sacral rectosigmoidectomy (Code 30); and (d) histology behavior is adenocarcinoma of the rectum (code as 8140/3). Our study included the following exclusion criteria: (a) race unknown (n=22); (b) marital status unknown (n=420); (c) T0, Tis, Tx (n=232); (d) Nx (n=136); (e) M1, Mx (n=1,705); (f) multiple primary tumor (n=1,989); (g) cause of death unknown (n=44); (h) size unknown (n=841); (i) number of lymph nodes unknown (n=43); and (j) number of positive lymph nodes unknown (n=1,577). Ultimately, 4,310 patients with lymph- node- positive rectal cancer from the SEER database were included in our study based on inclusion and exclusion criteria and analyzed (**Figure 1**).

### 2.2 An explanation of the variables and endpoints

The SEER database included several components of variables namely, characteristics of the population (age at diagnosis, sex, race, and marital status), characteristics of tumors (histology, T stage, N stage, tumor size, number of lymph nodes, and number of positive lymph nodes), information on treatment (chemotherapy, radiotherapy, and surgical treatment), and survival information (months of survival and CSS).

In the column “RX Summ-Surg Prim Site (1998 +),” the surgical method was chosen. Anterior resection, Hartmann’s operation, low anterior resection (LAR), and trans-sacral rectosigmoidectomy were coded as 30. We converted continuous variables such as age, tumor size, number of lymph nodes examined, and number of positive lymph nodes examined into categorical variables: age (≤60, 61–78, and ≥78), tumor size (<3 cm, 3–5cm, and >5 cm), lymph nodes examined (<11, 11–20, 21–30, and >30), and positive lymph nodes examined (1, 2–10, and >10). Other variables included (1) sex (male, female), (2) race [American Indian/Alaska Native (AIAN), Asian or Pacific Islander (API), black, white], (3) marital status [married, others (divorced/separated, single, and widowed)], (4) T stage (T1, T2, T3, T4), (5) N stage (N1, N2), (6) radiotherapy (no/unknown, yes), and (7) chemotherapy (no/unknown, yes).

In SEER, CSS is classified as a potentially fatal cause of rectal cancer, according to the cause -specific death classification column. In this study, CSS is defined as the interval between initial cancer diagnosis and specific death from rectal cancer.

### 2.3 Statistical analysis

Of the patients, 70% were randomly assigned to the development cohort and 30% to the validation cohort. The selected variables in the development cohort were evaluated by univariate Cox regression analysis, and variables that had statistical difference (p-value<0.05) were included in multivariate Cox regression analysis. In Cox regression analysis, all results were presented as hazard ratios (HR) and their 95% confidence intervals (CIs). It was applied to the development cohort to build nomograms and a system of risk classification. The validation cohort underwent internal validation.

We used the concordance index (C-index), calibration curves, and receiver operating characteristics curve (ROC) to verify the nomogram. The C-index was used to reflect the performance and prediction accuracy of the nomogram. In our study, calibration plots (1,000 bootstrap resamples) were plotted for 3 and 5 years to compare predicted and observed CSS. In the calibration diagram, the 45-degree line represents the actual results of the model. Generated ROC curves were based on specificity and sensitivity of the nomogram. Based on the cutoff values of the development cohort’s total nomogram scores, patients were divided into low-, intermediate-, and high-risk groups. In order to compare the CSS of patients in different risk groups, Kaplan–Meier curves and log-rank tests were used.

We extracted the data using SEER*Stat software version 8.4.0.1. A comparison of baseline data between the development cohort and validation cohort was conducted using SPSS 25.0 (IBM Corp, Armonk, NY). Cox regression analyses based on univariate and multivariate variables, plotting nomogram, C-index, calibration plots, ROC curves, and Kaplan–Meier curves were generated using R version 4.2.0 and related packages. In the X-Tile version 3.6.1, the cutoff value of age and total score were calculated. When p-value < 0.05, the differences were statistically significant.

## 3 Results

### 3.1 Baseline patient characteristics

We included a total of 4,310 patients with lymph- node- positive rectal cancer in our study, including 3,017 in the development cohort and 1,293 in the validation cohort. Of the patients included in the study, 2,503 (58.1%) were male, 3,419 (79.3%) were white, 2,684 (62.3%) were married, and 3,202 (74.3%) were had stage T3. A total of 2,152 (49.9%) patients had tumors with a diameter of 3–5 cm. Most patients (n=3,414, 79.2%) received chemotherapy and 2,999 (69.6%) received radiotherapy. The following graph shows the baseline demographic and clinical characteristics of patients in both the development cohort and the internal validation cohort. There was no significant difference between the two groups in terms of baseline data (**Table 1**). Upon completion of follow-up, a total of 1,600 (37.1%) patients died of rectal cancer, including 1,147 (38.0%) in the development cohort and 453 (35.0%) in the validation cohort.

**Table 1:** Baseline demographic and clinical characteristics of lymph- node-positive rectal cancer patients in the development and validation cohorts.

Characteristics	Total cohort n=4,310 N (%)	Development cohort n=3,017 N (%)	Validation cohort n=1,293 N (%)	p-value
Age				0.930
≤60	2,213 (51.3%)	1,549 (51.3%)	664 (51.4%)	
61–78	1,585 (36.8%)	1,113 (36.9%)	472 (36.5%)	
>78	512 (11.9%)	355 (11.8%)	157 (12.1%)	
Sex				0.888
Female	1,807 (41.9%)	1,267 (42.0%)	540 (41.8%)	
Male	2,503 (58.1%)	1,750 (58.0%)	753 (58.2%)	
Race				0.852
AIAN	33 (0.8%)	23 (0.8%)	10 (0.8%)	
API	515 (11.9%)	357 (11.8%)	158 (12.2%)	
Black	343 (8.0%)	247 (8.2%)	96 (7.4%)	
White	3,419 (79.3%)	2,390 (79.2%)	1,029 (79.6%)	
Marital status				0.773
Married	2,684 (62.3%)	1,883 (62.4%)	801 (61.9%)	
Others	1,626(37.7%)	1,134 (37.6%)	492 (38.1%)	
T stage				0.420
T1	183 (4.2%)	118 (3.9%)	65 (5.0%)	
T2	643 (14.9%)	454 (15.0%)	189 (14.6%)	
T3	3,202 (74.3%)	2,247 (74.5%)	955 (73.9%)	
T4	282 (6.5%)	198 (6.6%)	84 (6.5%)	
N stage				0.703
N1	2,755 (63.9%)	1,923 (63.7%)	832 (64.3%)	
N2	1,555 (36.1%)	1,094 (36.3%)	461 (35.7%)	
Chemotherapy				0.987
No/Unknown	896 (20.8%)	627 (20.8%)	269 (20.8%)	
Yes	3,414 (79.2%)	2,390 (79.2%)	1,024 (79.2%)	
Radiation				0.925
No/Unknown	1,311 (30.4%)	919 (30.5%)	392 (30.3%)	
Yes	2,999 (69.6%)	2,098 (69.5%)	901 (69.7%)	
Tumor size				0.194
<3 cm	1,035 (24.0%)	716 (23.7%)	319 (24.7%)	
3–5 cm	2,152 (49.9%)	1,491 (49.4%)	661 (51.1%)	
>5 cm	1,123 (26.1%)	810 (26.8%)	313 (24.2%)	
Lymph nodes				0.501
<11	952 (22.1%)	652 (21.6%)	300 (23.2%)	
11–20	2,291 (53.2%)	1,601 (53.1%)	690 (53.4%)	
21–30	786 (18.2%)	563 (18.7%)	223 (17.2%)	
>30	281 (6.5%)	201 (6.7%)	80 (6.2%)	
Positive lymph nodes				0.861
1	1,481 (34.4%)	1,041 (34.5%)	440 (34.0%)	
2–10	2,571 (59.7%)	1,799 (59.6%)	772 (59.7%)	
>10	258 (6.0%)	177 (5.9%)	81 (6.3%)	

AIAN, American Indian/Alaska Native; API, Asian or Pacific Islander; * Statistical significance (p < 0.05).

### 3.2 Finding and identifying predictive factors

The predictive power of the factors was evaluated using Cox regression analysis. In the univariate Cox regression analysis of the development cohort, age, race, marital status, T stage, N stage, chemotherapy, radiation, tumor size, lymph nodes, and positive lymph nodes were significantly different (p < 0.05). As a result of the multivariate Cox regression analysis, we obtained the following results: age, race, T stage, tumor size, lymph nodes, and positive lymph nodes had a great influence on prognosis. In terms of age, younger age is associated with better prognosis; the age>78 contributed to worse survival. In terms of race, American Indian/Alaska Native (AIAN) people have better survival, and Asian or Pacific Islander (API) people have worse survival. For T stage, T1 contributes to better survival, and T4 contributes to worse survival. As for lymph nodes and positive lymph nodes, a positive correlation was found between the number of lymph nodes examined and patient prognosis, while a negative correlation was found between the number of positive lymph nodes and patient prognosis. In the final analysis, nine predictors, including age, race, marital status, T stage, N stage, chemotherapy, tumor size, lymph nodes, and positive lymph nodes, were identified as independent predictors of CSS (**Table 2**).

**Table 2:** Univariate Cox regression analysis and multivariate Cox regression analysis of CSS in the development cohort.

Characteristics	Univariate analysis HR (95%CI)	p-value	Multivariate analysis HR (95%CI)	p-value
Age				
≤60	Ref.		Ref.	
61–78	1.554(1.369–1.765)	<0.001*	1.522(1.337–1.732)	<0.001*
≥78	3.571(3.021–4.221)	<0.001*	3.216(2.667–3.877)	<0.001*
Sex				
Female	Ref.			
Male	1.069(0.950–1.203)	0.268		
Race				
AIAN	Ref.		Ref.	
API	0.390(0.232–0.656)	<0.001*	0.416(0.246–0.702)	0.001*
Black	0.647(0.384–1.090)	0.102	0.690(0.408–1.166)	0.166
White	0.427(0.260–0.700)	<0.001*	0.478(0.291–0.787)	0.004*
Marital status				
Married	Ref.		Ref.	
Others	1.422(1.265–1.599)	<0.001*	1.157(1.024–1.306)	0.019*
T stage				
T1	Ref.		Ref.	
T2	1.185(0.754–1.860)	0.462	1.169(0.743–1.840)	0.499
T3	2.400(1.587–3.631)	<0.001*	2.153(1.412–3.282)	<0.001*
T4	5.008(3.209–7.818)	<0.001*	4.213(2.671–6.646)	<0.001*
N stage				
N1	Ref.		Ref.	
N2	1.616(1.439–1.816)	<0.001*	1.272(1.102–1.468)	0.001*
Chemotherapy				
No/Unknown	Ref.		Ref.	
Yes	0.546(0.478–0.624)	<0.001*	0.813(0.682–0.969)	0.021*
Radiation				
No/Unknown	Ref.		Ref.	
Yes	0.657(0.581–0.742)	<0.001*	0.874(0.748–1.021)	0.088
Tumor size				
<3cm	Ref.		Ref.	
3–5cm	1.400(1.198–1.636)	<0.001*	1.126(0.957–1.323)	0.148
>5cm	1.683(1.421–1.993)	<0.001*	1.327(1.110–1.587)	0.002*
Lymph nodes				
<11	Ref.		Ref.	
11–20	0.736(0.641–0.845)	<0.001*	0.691(0.600–0.797)	<0.001*
21–30	0.706((0.591–0.844)	<0.001*	0.539(0.446–0.651)	<0.001*
>30	0.581(0.441–0.765)	<0.001*	0.414(0.311–0.552)	<0.001*
Positive lymph nodes				
1	Ref.		Ref.	
2–10	1.586(1.386–1.815)	<0.001*	1.458(1.250–1.702)	<0.001*
>10	2.863(2.296–3.570)	<0.001*	2.804(2.143–3.671)	<0.001*

AIAN, American Indian/Alaska Native; API, Asian or Pacific Islander; * Statistical significance (p < 0.05).

### 3.3 Construction of nomogram and validation of the discrimination capability

**Figure 2:**
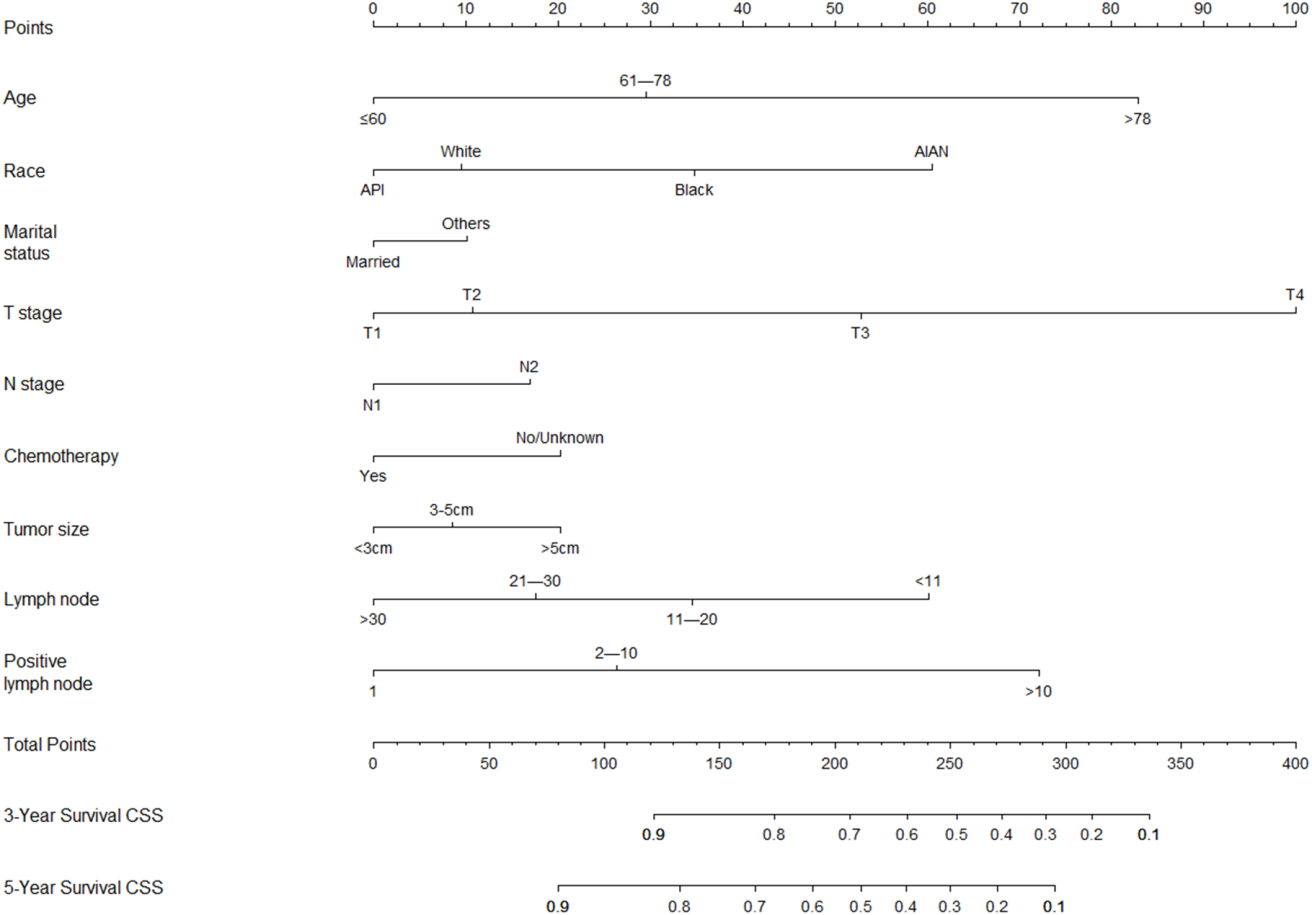
Nomogram predicting 3- and 5-year CSS probabilities for lymph-node- positive rectal cancer patients after radical proctectomy.

Based on these CSS prognostic factors, an algorithm for predicting 3- and 5-year CSS in lymph- node- positive rectal cancer patients had been developed and presented virtually as a nomogram (**Figure 2**). According to the nomogram, we observed that young age, receiving chemoradiotherapy, small tumor size, more lymph nodes (positive lymph nodes and negative lymph nodes), and fewer positive lymph nodes were associated with better prognosis of rectal cancer. Patients with stage T4 had the worst prognosis. The results obtained by the nomogram were in agreement with those obtained by previous studies (14–18).

**Figure 3:**
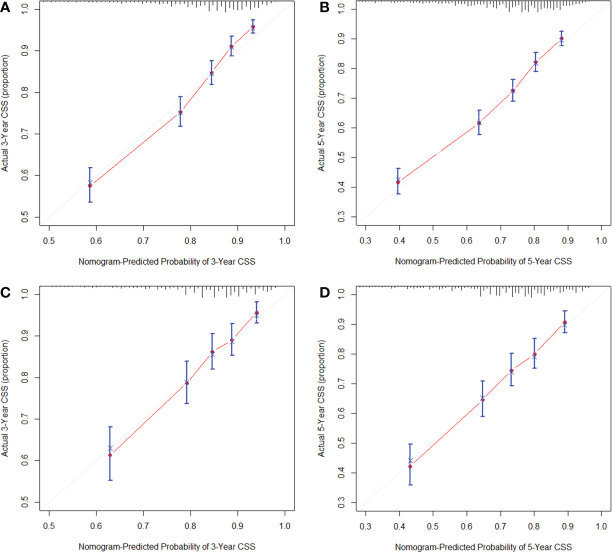
Calibration plots of the nomogram for 3-year **(A)** and 5-year CSS **(B)** in the development cohort; calibration plots of the nomogram for 3-year **(C)** and 5-year CSS **(D)** in the validation cohort.

**Figure 4:**
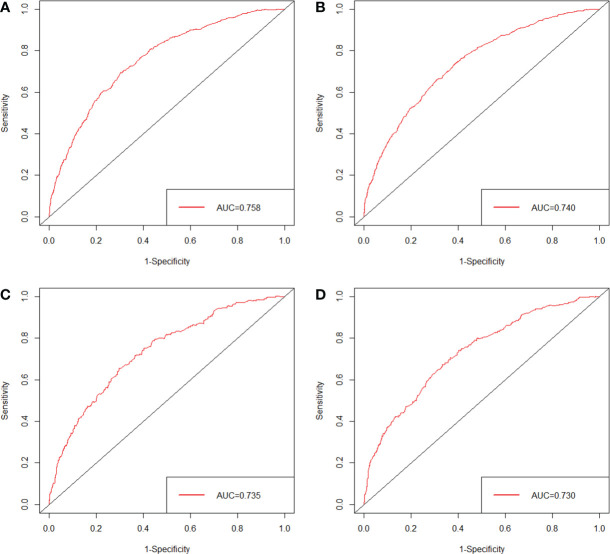
ROC of the nomogram predicting CSS for 3 years **(A)** and 5 years **(B)** in the development cohort; ROC of the nomogram predicting CSS for 3 years **(C)** and 5 years **(D)** in the validation cohort.

The nomogram was validated in the internal validation cohort. The nomogram C-index was 0.702 (95% CI, 0.687–0.717) in the development cohort and 0.690 (95% CI, 0.665–0.715) in the validation cohort. In both the development cohort and the validation cohort, the calibration curves showed good agreement between predicted results and actual observations for 3- and 5-year CSS (**Figure 3**). The AUCs were 0.758 and 0.740 for 3- and 5-year CSS in the development cohort, respectively (**Figures 4A, B**), and 0.735 and 0.730 for 3- and 5-year CSS in the validation cohort, respectively (**Figures 4C, D**), respectively. All these results demonstrate the good performance and application of our nomogram.

### 3.4 Risk classification system

**Figure 5:**
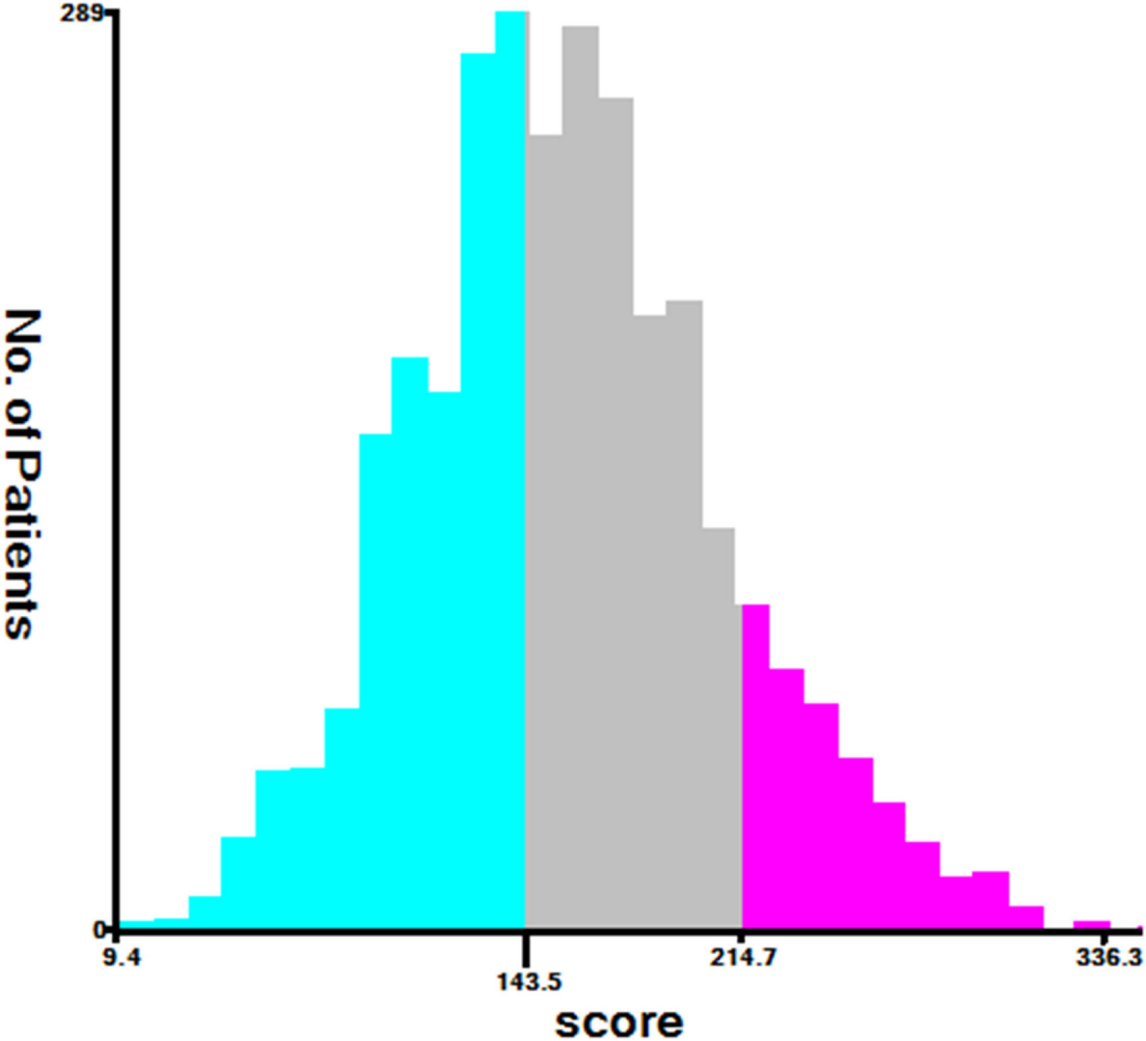
Range of risk stratification based on nomogram total score.

**Figure 6:**
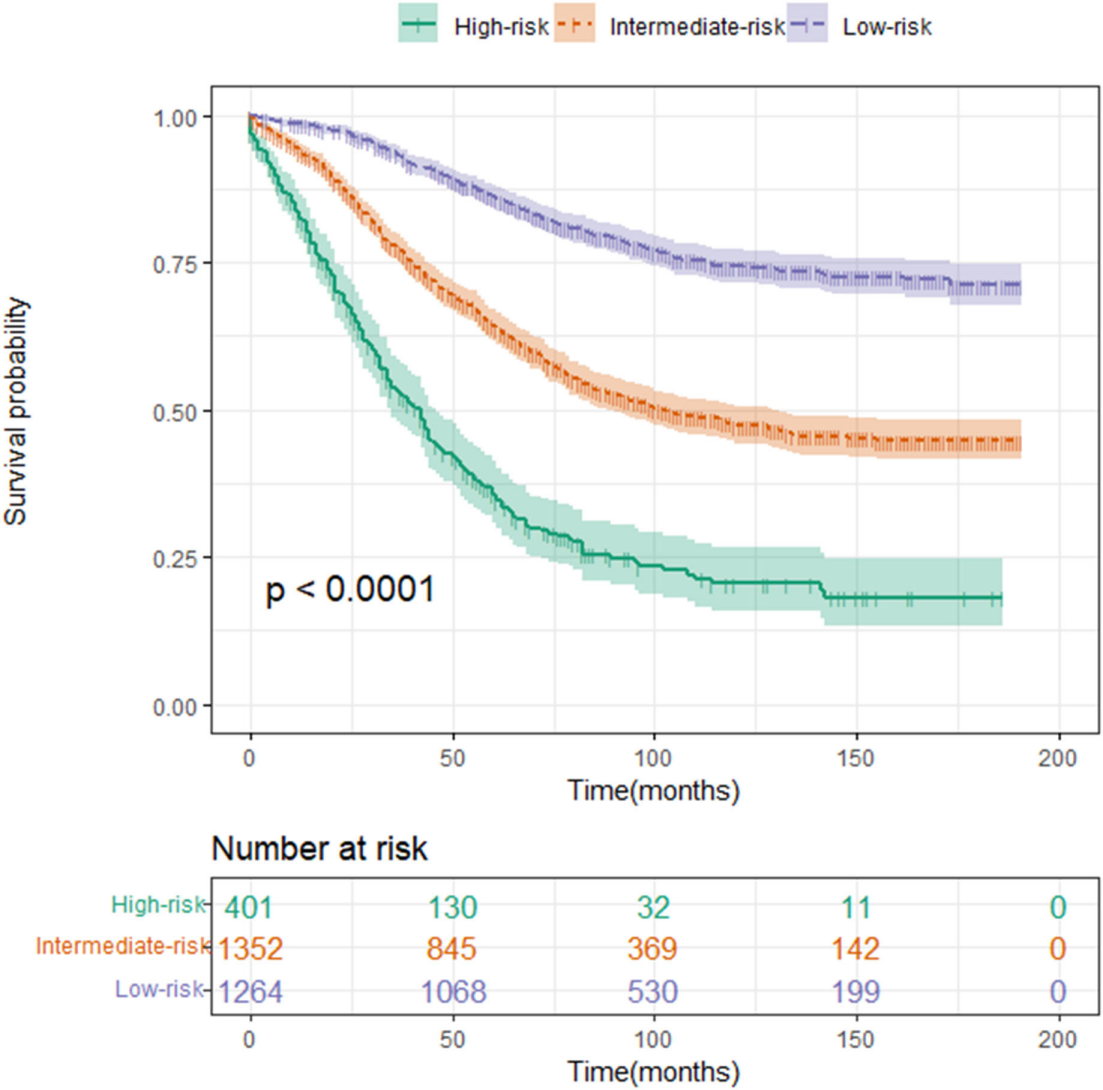
Kaplan–Meier curve of CSS for risk classification based on the nomogram scores in the development cohort. Low-risk group (score<143.54); intermediate-risk group (score=143.54–214.75); and high-risk group (score >214.75).

All factors in the nomogram that we built in the study were scored between 0 and 100 based on how much they contributed to the nomogram (**Table 3**). As part of the nomogram generation, based on the cutoff value of the total score in the development cohort, we developed a risk stratification system (**Figure 5**). All patients in the development cohort were divided into low-risk group (1,264/3,017, score<143.54), intermediate-risk group (1,352/3,017, score = 143.54–214.75), and high-risk group (401/3,017, score >214.75). Kaplan–Meier analysis showed a significant difference in CSS between the low-, intermediate-, and high-risk groups (p<0.05) (**Figure 6**).

**Table 3:** Nomogram scoring system.

Variables	Points		Variables		Points	Variables	Points	
Age			T stage			Tumor size		
≤60	0		T1		0	<3cm	0	
61–78	29.53		T2		10.67	3–5cm	8.56	
>78	82.91		T3		52.83	>5cm	20.25	
Race			T4		100	Lymph node		
AIAN	60.59		N stage			<11	61.12	
API	0		N1		0	11–20	34.55	
Black	34.74		N2		16.94	21–30	17.51	
White	9.44		Chemotherapy			>30	0	
Marital status			No/Unknown		20.25	Positive lymph node		
Married	0		Yes		0	1	0	
Others	10.13					2–10	26.35	
3-Year CSS probability			5-Year CSS probability			>10	72.16	
0.1	336.48		0.1		295.35			
0.2	311.50		0.2		270.36			
0.3	291.25		0.3		250.11			
0.4	272.20		0.4		231.06			
0.5	252.73		0.5		211.59			
0.6	231.44		0.6		190.30			
0.7	206.38		0.7		165.24			
0.8	173.66		0.8		132.52			
0.9	121.31		0.9		80.17			

## 4 Discussion

Most of the data for the previously proposed survival prediction models related to rectal cancer came from a single center with a limited study sample (19, 20). There are also models that incorporate limited predictors or evaluation metrics that are not readily available, and the clinical application of these models is greatly limited (21). Moreover, in some studies, the endpoints studied were limited to the prediction of overall survival (OS), and few studies have constructed a prediction of CSS (22). Furthermore, with the advancement of science and technology, new treatments for rectal cancer have been developed and improved, such as neoadjuvant chemotherapy (23, 24), targeted therapy (25, 26), and immunotherapy (27). These treatments may have changed the clinical outcomes of patients with rectal cancer. Therefore, existing prognostic analyses of patients with lymph- node- positive rectal cancer have been developed for a long time and may not meet today’s clinical needs (28). There is an urgent need to develop a predictive model applicable to patients with lymph-node-positive rectal cancer. Our study developed and validated a nomogram for lymph- node- positive rectal cancer patient after radical proctectomy 3- and 5- year CSS by analyzing baseline demographic and clinical characteristics of 4,310 patients. It provides clinical prognostic assessment for patients with lymph- node-positive rectal cancer.

During rectal cancer surgery, lymph nodes are dissected, and the extent of this procedure is closely related to the prognosis of the patient (29, 30). Studies have shown that an increase in detected lymph nodes (positive lymph nodes and negative lymph nodes) were associated with an increase in patient survival over the next 5 years, and increased lymph node positivity suggests a poor prognosis and recurrence and metastasis (31, 32). Our nomogram also evaluated patients based on the number of lymph nodes and the number of positive lymph nodes. A positive correlation was found between the number of lymph nodes examined and patient prognosis, while a negative correlation was found between the number of positive lymph nodes and patient prognosis.

As part of the study, we examined all available factors in the SEER database and their effects on the prediction of lymph- node- positive rectal cancer patients CSS. CSS can be prognosed by nine independent variables. The nine significant prognostic factors identified by Cox analysis were also found in previous studies (33–35). Although we found no new prognostic factors affecting rectal cancer, the prognostic factors that we identified were easily obtained from practical clinical work. Unlike some prediction models, our prognostic factors does not require a difficult-to-obtain gene test to predict prognosis (36, 37).With the help of a multivariate Cox model, we constructed and internally validated a nomogram that has relatively high accuracy and discriminative power. It included only significant variables associated with survival outcomes and did not lack accuracy. In modern times, cancer outcomes are predicted most often using the American Joint Committee on Cancer (AJCC) staging system. However, there are also great differences in the clinical outcomes of patients with rectal cancer at the same AJCC stage. It is evident from this that the AJCC staging system does not provide the best prognostic information. This AJCC staging system only considers T, N, and M stages, and does not consider other prognostic factors such as demographic characteristics and clinical treatment (38). Our nomogram contains not only clinicopathological information but also demographic characteristics and clinical treatment. In addition, another advantage of the nomogram over standard Cox regression models is that it provides individual survival outcome probabilities instead of a relative risk.

There were many patients involved in this study, and multiple factors were assessed for their impact on patients with lymph- node- positive rectal cancer. It has several advantages over other models. First, it has the advantage of being applicable to patients with lymph- node- positive rectal adenocarcinoma, which provides a better representation of the characteristics of this patient population. In addition, our nomogram takes into account both demographic information and clinicopathological information. In clinical practice, these are key indicators that can be easily accessed.

There are some limitations to this study. The first thing to note is that this is a retrospective study. Some patients whose variable information was unknown were excluded from the study based on strict inclusion and exclusion criteria. Therefore, potential selection bias may exist (39). Second, cancer information came from many different hospitals. The SEER database does not have specific step-by-step instructions, which may be biased due to the different operators and pathologists. Third, it is likely that this nomogram may not be applicable to patients outside North America, since the SEER database is mainly North American. Finally, the nomogram and risk classification system were well reflected and validated internally. However, the patients developed and validated were from the same database; the validation method was not perfect, and the prediction model still needed external validation.

## 5 Conclusion

In our study, a nomogram and novel risk classification system were constructed to predict 3- and 5-year CSS in lymph- node- positive rectal cancer patient after radical proctectomy. The nomogram has been proven to have not only good prognostic discrimination and survival predictive power but also good clinical decision-making power. This nomogram can be used for individualized postoperative survival prediction in patients with lymph- node- positive rectal cancer. Although we performed an internal validation, external validation of the lymph- node-positive rectal cancer dataset should be considered.

## Data availability statement

The original contributions presented in the study are included in the article/supplementary material. Further inquiries can be directed to the corresponding author.

## Ethics statement

Ethical review and approval was not required for the study on human participants in accordance with the local legislation and institutional requirements. Written informed consent for participation was not required for this study in accordance with the national legislation and the institutional requirements.

## Author contributions

All authors listed have made a substantial, direct, and intellectual contribution to the work and approved it for publication.
